# The Molecular Basis of PPAR Function

**DOI:** 10.1155/2010/510530

**Published:** 2011-03-08

**Authors:** Yaacov Barak, Chih-Hao Lee

**Affiliations:** ^1^Magee-Womens Research Institute, Department of OBGYN and Reproductive Sciences, University of Pittsburgh, 204 Craft Avenue, Pittsburgh, PA 15213, USA; ^2^Department of Genetics and Complex Diseases, Harvard University School of Public Health, 665 Huntington Avenue, Boston, MA 02115, USA

Over the past four and a half years, fifteen issues of PPAR Research have been published and an additional five are currently in various phases of production. Collectively, these issues have covered a large array of physiological functions of PPARs in health and disease, including their functions in diverse cell and tissue types, their roles in a host of clinical conditions, and their rich pharmaceutical potential. Yet, one crucial overarching selection seems to have escaped attention amid this exciting variety—the fundamental molecular mechanisms that underlie PPAR action. PPARs are first and foremost transcription factors that execute intricate regulation of gene networks, and are in turn subject to complex fine-tuning and control. Inevitably, thorough understanding of their cellular and organismal functions and full capitalization on their pharmaceutical power require detailed understanding of these molecular underpinnings. 

 While the list of potential subplots is far too long to fit into a single issue, the current issue has begun to fill this coverage gap. Three review articles and one research manuscript tackle the subject of PPAR target genes. First is a comprehensive review of PPAR*α* target genes by Rakhshandehroo et al. Although the primary focus of their review is hepatic targets, many of the discussed features are applicable to the physiology of PPAR*α* in other tissues. Next, Bugge and Mandrup review two complementary aspects of PPAR*γ*-regulated genes. The first concerns the role of the N-terminal ligand-independent activation domain, also known as the A/B or AF-1 domain, in determining PPAR subtype specificity and in modulating target activation in adipocytes. The second focuses on recent insights into genomewide PPAR*γ* actions in adipocytes and macrophages gained from chromatin immunoprecipitation studies. In the third review, Costa et al. discuss population-wide sequence variations of PPAR*γ* and their potential impact on target gene expression, and review the application of massive parallel sequencing for the identification of PPAR*γ* cistromes (the collection of genomic sequences bound by PPAR*γ*) and transcriptomes. In the second section of this issue, the research article by Le Bouter et al. analyzes gene expression changes in adipose tissue, liver, and muscle of leptin receptor deficient diabetic obese mice treated with the PPAR*γ* agonist and insulin sensitizer rosiglitazone, in order to establish the molecular basis of its therapeutic physiological effects. 

Two papers by Viswakarma et al. and Pyper et al., one a review and the other a research manuscript, concern transcription cofactors of PPARs. Their thorough review summarizes no less than twelve types of PPAR coactivators, which, ironically, do not account for the fullest gamut of known PPAR coactivators. Nothing is better to illustrate the complexity of this ever-growing subplot than this group's accompanying research article, which describes the identification and initial characterization of yet a new PPAR coactivator, a subunit of the mediator complex termed PRIC295.

In the course of executing their physiological roles, PPARs are both regulated by and intersect with diverse signaling pathways. Three review articles touch on three of these many types of interactions. Charoensuksai and Xu review the effects of circadian signaling on the expression and activities of PPARs, Moreno et al. summarize the interactions between nutrient signaling and PPARs, whereas Takada et al. expand on the interaction of PPAR*γ* with downstream effectors of cytokine and Wnt signaling in mesenchymal stem cells.

While this volume is a solid start, it did not go the full distance and many of the molecular bases of PPAR function remain to be reviewed. Some topics, such as the structure-function relationships of PPARs or their posttranslational regulation won no coverage in this issue. Among the touched upon topics, some additional reviews are crucial for rounding out the coverage, such as target genes of PPAR*β*/*δ* or additional signaling crosstalk of PPARs. And so, it would be great to see a fresh team of guest editors pick the topic up where we left off and cover the next expanse of this literature gap. 

**Yaacov Barak** **Yaacov Barak** **Chih-Hao Lee** **Chih-Hao Lee**

